# *Toxoplasma gondii*: Preventive and therapeutic effects of morphine and evaluation of treatment parameters of tachyzoites and infected macrophages *in vitro* and in a murine model 

**DOI:** 10.17179/excli2019-1961

**Published:** 2020-04-20

**Authors:** Leila Zaki, Fatemeh Ghaffarifar, Zohreh Sharifi, John Horton, Javid Sadraei

**Affiliations:** 1 Department of Parasitology, Faculty of Medical Sciences, Tarbiat Modares University, Tehran, Iran; 2 Blood Transfusion Research Center, High Institute for Research and Education in Transfusion Medicine, Tehran, Iran; 3 Tropical Projects, Hitchin, United Kingdom

**Keywords:** Toxoplasma gondii, morphine, In vivo, BALB/c mice

## Abstract

Common medicines for the treatment of toxoplasmosis have limited efficacy and unwanted side effects. Opiates can effect both innate and cell-mediated immunity and stimulate the immune responses in different parasitic infections. In this work, preventive and therapeutic effects of morphine were evaluated on the tachyzoites of *Toxoplasma gondii* and infected macrophages *in vitro* and in a murine model. Different concentrations of morphine (0.1 and 0.01 μg/ml) were evaluated on mortality rate of *T. gondii* by direct counting after 3 and 24 hours. The cytotoxic and apoptotic effects of these drugs were measured by the MTT assays and flow cytometry analysis, respectively. The same procedures were assessed in *T. gondii*-infected macrophages. The parasite loads were determined using quantitative polymerase chain reaction (qPCR). For in* vivo* assessment, BALB/c mice treated with morphine before or after infection with tachyzoites. The survival rate of animals, parasite load in the spleen, and the IFN-γ and IL-4 cytokines levels were measured. Morphine was effective on tachyzoites of *T. gondii* and had a reverse relationship with its concentration. The results of flow cytometry showed that the toxic effects of morphine on tachyzoites after 3 hours was not statistically significant (*p*<0.05). Also, apoptosis in infected MQs rose with a decreasing concentration of morphine. The parasitic load in MQs treated with morphine before infection was lower than that in cells treated after infection and the differences were statistically significant (*p*<0.01). In mice that received morphine before infection, survival rate, parasite load and the IFN-γ level were significantly lower than in mice treated after infection (*p*<0.01). The results of this study have shown that morphine in the pre-treatment group had higher anti-*Toxoplasma* activity than morphine in post-treatment *in vitro* and in murine model.

## Abbreviations

***T. gondii*** *Toxoplasma gondii*

**MQs** macrophages

**S** sulfadiazine

**PYR** pyrimethamine

**M** morphine

**IFN-γ** interferon-γ

**IL-4** interleukin-4

## Introduction

Toxoplasmosis is a common parasite disease, caused by a ubiquitous intracellular protozoan parasite known as *Toxoplasma gondii (T. gondii*) that infects up to one third of the people around the globe (Dubey, 2008[5]; Montoya and Liesenfeld, 2004[18]). This parasite is transmitted through consumption of *T. gondii* tissue cysts contained in raw/undercooked meats, by ingestion of food or water that is contaminated by mature oocysts and congenitally from pregnant mothers to the fetus. Occasionally it occurs in individuals who received blood transfusions and organ transplantation (Fallahi et al., 2018[7]; Gharavi et al., 2011[13]). It is likely that chronic toxoplasmosis has a relationship with some diseases such as type 2 diabetes mellitus, mental illnesses, schizophrenia and bipolar disorder (Foroutan et al., 2019[10]). In addition, seronegative pregnant women and patients suffering from immune deficiency disorders such as immunodeficiency virus‒positive individuals and with some types of malignancy appear to be most susceptible to congenital and fatal toxoplasmosis if not treated (Fishman, 2013[8]; Wang et al., 2017[30]). 

Despite of great advances, establishing an effective therapeutic scheme for prevention and control of toxoplasmosis in both humans and animals still remains a great challenge for researchers to select new anti-*Toxoplasma* drugs with specific activity against the parasite. The most common drugs for treatment of toxoplasmosis are combinations of pyrimethamine (PYR) and sulfadiazine (S) that have limited efficacy and unwanted side effects (Montoya and Gomez, 2016[19]) Additionally, these drugs are unable to eliminate the encysted parasites within infected hosts. Hence, identification of appropriate and effective drugs that can act as adjunctive therapy are of great priority and are urgently required to enhance efficacy and reduce toxicity (Antczak et al., 2016[2]; Foroutan et al., 2018[9]).

Although morphine and other opioids have critical roles as antifibrotic, anti-inflammatory and antitumor agents, antimicrobial activities are also important effects of these drugs (Dinda et al., 2005[4]).

Opioid medicines can effect ordinary functions of immune cells such as macrophages and polymorphonuclear leukocytes, which produce a variety of factors necessary for a functional immune response in humans and experimental animals (Fuggetta et al., 2005[12]; Singal and Singh, 2005[27]). On the other hand, the ability of morphine to provoke the immune system is relied up on in the observed dose-dependent impacts of this drug (Fuggetta et al., 2005[12]). Administration of high doses of morphine trigger the immune response to defense against infectious disease (Wang et al., 2005[29]), but administration of low doses of morphine suppresses immune responses to increase susceptibility to infection (Jabari et al., 2019[14]; Singal et al., 2002[26]; Singh et al., 1994[28]). It is well known that an extracellular agonist binds to G-protein coupled receptors to cause cellular hyperpolarization (Sharp et al., 1998[25]).

Until now, no previous studies have been conducted to investigate preventive and therapeutic effects of morphine on the tachyzoites and experimental infected macrophages by *Toxoplasma gondii*. Hence, in this work, we determine *in vitro* and *in vivo* activities of the morphine against *Toxoplasma gondii*.

## Materials and Methods

### In vitro assay

#### Parasite culture and harvesting

RH strains of *T. gondii* were obtained from the Parasitology Department of Tarbiat Modares University. In order to increase the number of parasites, intraperitoneal passages in mice were carried out with 1×10^6^ parasites, and then the ascitic fluid collected 72 h after infection. Tachyzoites were washed with cold phosphate-buffered saline (PBS) and centrifuged at 2000 RPM for 10 min at 4 °C and their concentration was determined by trypan blue exclusion in hemocytometric chamber.

#### Preparation of drugs

In this work, 1 mg of morphine sulfate powder (Temad Co, Teheran, Iran) was dissolved in 1 ml normal saline 0.9 % and then various concentrations (100, 10, 1, 0.1 and 0.01 μg/ml) of morphine were prepared from the stock solution with 1000 μg/ml concentration. Sulfadiazine and pyrimethamine (Sigma Aldrich, St. Louis, USA) were used at concentrations of 40 and 1 μg/ml, respectively. 

#### Cytotoxicity assay on tachyzoites

In order to evaluate the parasite survival, tachyzoites of *T. gondii* were seeded at 100 µL/well in 96-well plates (cell suspensions 2 × 10^6^ cells/mL in RPMI 1640 medium enriched with 10 % FBS), in the presence of various concentrations (100, 10, 1, 0.1 and 0.01 μg/ml) of morphine in triplicate and were kept at a temperature of 37 °C for 6 and 24 hours separately (wells without drug were used as negative control groups). Morever, S (40 µg/mL) plus PYR (1 µg/mL) were considered as positive control groups. After incubation, the number of treated and untreated parasites at the different drug doses was estimated by direct counting in a hemocytometer (Neubauer chamber) using a phase contrast microscope and the data was analyzed using Graph pad Prism version 8.0.1 software for determination of the parasite survival for each well with the results compared to the control groups. 

#### Cultivation and collection of infected macrophage cells

Initially*,* RAW 264.7 macrophage cells (cell line from mouse BALB/c monocyte macrophage) were cultured in a 75 cm^2 ^cell culture flask containing RPMI 1640 medium (Gibco, Germany) with 10 % heat inactivated FCS, 100 U/ml penicillin, and 100 µg/ml streptomycin, and then incubated in a humidified atmosphere of 95 % air and 5 % CO_2_ at 37 °C. 

For evaluation of therapeutic effects, these MQs were trypsinized and embedded to 24-well microplates by 1 ml per well at 1×10^5 ^cells/well in 10 % FCS RPMI 1640 medium at a temperature of 37 °C with 5 % CO_2_ for 24 h. MQs were then infected with *T. gondii* at a final concentration of 2×10^5^ cells/ml and the plates maintained under the previous conditions. Six hours after infection, the cells were washed twice with cold phosphate buffer saline (PBS) to remove unadhered MQs from the wells and fresh culture medium was supplemented with the different concentrations of drugs. Control wells contained parasite-infected MQs without drug. After 24 h incubation, the cells were collected and transferred into 1.5 mm DNase/RNase-free tubes and kept at a temperature of -70 °C until used.

For evaluation of preventive effects, the cultured MQs (1 ml, 1×10^5^ cell ml^−1^) were added to 24-well microplates and incubated for a period of 24 h under the same conditions. Next, supernatant was discarded and unadhered cells were removed. For treatment, 1 ml culture media containing different concentrations of drug were added to the wells. Incubation of the treated MQs was carried out for 24 h again. The adhered MQs were infected using tachyzoites of *T. gondii*, at a ratio of 2:1 (parasite/MQs). The plate was then incubated in 37 °C with 5 % CO_2_. The rest of the stages were done as mentioned above. 

#### Assessment of parasite load of infected macrophages

In both models (pre-treatment and post-treatment), the RNA extraction from MQs was accomplished by the Qiagen RNA isolation kit following the manufacturer's protocol (RNeasy Mini Kit, Qiagen Company). The purity and quantity of the extracted RNA was determined with Nanodrop device (Roche-Germany). After that, cDNA was synthesized using the cDNA Synthesis Kit (Quanti Tect Reverse Transcription Kit, Qiagen Company) with 2 μg of total RNA. The standard curve samples were obtained by 6 fold-dilutions ranging from 2×10^1^ - 2×10^6^ parasites (initial concentration of 10^7^ tachyzoites). The Threshold Cycle (Ct) was calculated for these standard curves through SYBR-green real-time PCR using RE gene primers (5′-AGG GAC AGA AGT CGA AGG GG -3′ for the forward and 5′-GCA GCC AAG CCG GAA ACA TC -3′ for the reverse), and then the parasite loads were evaluated for all experimental samples. Amplification reactions were conducted in final volumes of 25 μL, comprising 12.5 μl of SYBR Green PCR Master Mix (QIAGEN), 2 μl of template cDNA, 1 μl of each primer and 8.5 μl of nuclease-free water. The quantitative real-time PCR conditions consisted of an initial step at 95 °C for 15 min, followed by 40 cycles of 95 °C for 15 s, and 60 °C for 15 s, and a final extension step at 72 °C for 15 s. Eventually, the temperatures of the melt curves were adjusted from 72 to 95 °C to ensure the specificity of the amplification products.

#### Toxic effects of morphine upon the uninfected macrophages

In the present study, MTT assay was taken for assessing uninfected MQs viability and cytotoxicity. In brief, MQs (2 × 10^4^ cells/well, 100 µl) were seeded in the 96-well microtiter plate that supplemented with complete RPMI 1640 medium as triplicates. After adhering the cells to the bottom of the wells, the plate was washed twice and then 100 µl of fresh culture medium was added to each well, containing different concentrations of morphine (from 100 to 0.01 μg/ml). After 24 h incubation (37 **°**C, 5 % CO_2_), 20 μL of solution of MTT (0.5 µg/ml) was added into each well and further incubated for 3-4 h. The plate was centrifuged for 10 minutes at 3000 g and then the contents of each well was discharged slowly and replaced with 100 μL of dimethyl sulfoxide (DMSO) to transform the yellow tetrazolium salt to insoluble blue formazan dye in healthy cells. Optical density (OD) of each well, using an ELISA reader was read within 30 minutes at 570 nm. The following formula was taken to assess the percentage of cell viability in comparison to controls.

% viable cells = (drug well absorption – blank well absorption / control well absorption – blank well absorption)×100.

#### Apoptotic effects of morphine on the tachyzoites and infected macrophages by flow cytometry

Annexin V-FITC Apoptosis Detection Kit (Bio Vision, Palo Alto, USA) was used to identify both necrosis and apoptosis in cells. In brief, in 12-well plates, 5 × 10^5^ tachyzoites of *T. gondii* were cultured in the presence of various concentrations of morphine and incubated for 6 h at a temperature of 37 °C in 5 % CO_2_ and 95 % humidity. Also, infected MQs exposed to different drug compounds were incubated for 24 h as described above. The cells were transferred to 1.5 ml microtubes and centrifuged at 3000 rpm for 5 min. The supernatant was then drained off and replaced with 500 μL binding buffer. Afterwards, 5 μL of annexin V and 5 μL of propidium iodide (PI) were added to cell pellets, and then incubated in the dark at a temperature of 24 ± 2 °C for 5 minutes. Using FACSCaliber (BD Biosciences), absorption of annexin-v was estimated and finally analyzed by CellQuest software.

### In vivo assay

#### Allocation and treatment

Sixty BALB/C mice (female, 6-8 weeks old) with a mean weight of 18-20 g were purchased from the Royan Institute (Tehran, Iran) and housed under standard laboratory conditions at an ambient temperature of 20 to 25 °C and relative humidity ranging from 55 ± 65 %. All mouse experiments were performed in accordance with approved protocols by the Medical Ethics Committee of Tarbiat Modares University of Iran for the care and use of laboratory animals. In the present study, these mice were grouped into 6 sets of 10 animals in each cage. One group before infection with 1 ×10^4^ *T. gondii* tachyzoites was treated with M (1 mg/kg, once a week) for 3 weeks. The rest of the groups after infection with the same dose were treated with M (1 mg/kg, once a week), S (40 mg/kg/day) + PYR (1 mg/kg/day), S (40 mg/kg/day) + PYR (1 mg/kg/day) + M (1 mg/kg, once a week). Healthy mice just received PBS as a negative control group while a positive control group was infected with tachyzoites and was not treated.

#### Survival assay

To evaluate the survival rate of the mice, five mice in each group after infection with 10^4^ live tachyzoites of strain RH *T. gondii*, were monitored daily and the mortality rate was documented for each group until death or day 10 post-inoculation.

#### Evaluation of parasite load of spleen tissues by quantitative real time PCR

After treatment as above and as soon as death occurred, five animals from each of the study groups were humanely killed, the spleen tissues were carefully removed and immersed in 5 ml of sterile phosphate buffer solution (PBS; pH 7.4). In order to estimate the parasite load using quantitative real time PCR targeting the RE gene, approximately 30 mg of spleen tissue from each mouse were transferred into 1.5 mm DNase/RNase-free tubes and homogenized with a mortar and pestle. The remaining spleen tissues were used for cytokine evalution. Assessment of parasite load of spleen tissues was carried out according to the protocol described above.

#### Cytokine level measurement

Following destruction of the spleens, lymphocytes were extracted and cultured (1 ml, 5×10^6^ cell ml^−1^) in 24-well plates in the RPMI 1640 containing 10 % FBS (Gibco-BRL, France), penicillin (100 unit ml^−1^), and streptomycin (100 μgml^−1^) (Sigma, Germany). For preparing Toxoplasma Lyzate Antigen, 2×10^9^ tachyzoites of RH strain of *T. gondii* were passed through filter membranes of 3 μm pore size and centrifuged at 4 °C, 3 times for 15 min. Then, the supernatant was discarded without disturbing the pellet and debris cells were removed. Phenylmethylsulphonyl fluoride (PMSF) as a protease inhibitor was added to a final concentration of 5 mM and the cell pellet was freeze-thawed 5 times alternatively in liquid nitrogen and water bath of 37 °C until no intact parasites were visible microscopically. After that, the protein content of TLA was measured by Bradford method and kept at -20 °C. To stimulate cells, 50 μg/ml *Toxoplasma* Lyzate Antigen were added to wells and incubated in a humidified atmosphere of 95 % air and 5 % CO_2_ at 37 °C for 3 days. After incubation, the supernatant was harvested and stored frozen at -80 °C until used for serologic evaluation. Assessment of IL4 and IFN-γ cytokines were performed according to manufacturer's recommended procedure (MabTag, Friesoythe, Germany) using ELISA.

#### Data analysis and statistics

All statistical analyses were carried out using IBM SPSS Statistics Version 21 and Graph Pad Prism version 8.0.1. The results were recorded and described by mean, standard deviation (SD), and 95 % confidence interval (CI). Analysis of variance (one-way-ANOVA) was performed to determine statistical significance. Parametric tests such as unpaired samples t-test were used for the comparisons of the data between the treatment and control groups and the level of significance was assessed at 5 % (p < 0.05).

## Results

### Tachyzoite viability test

**Figure 1:**
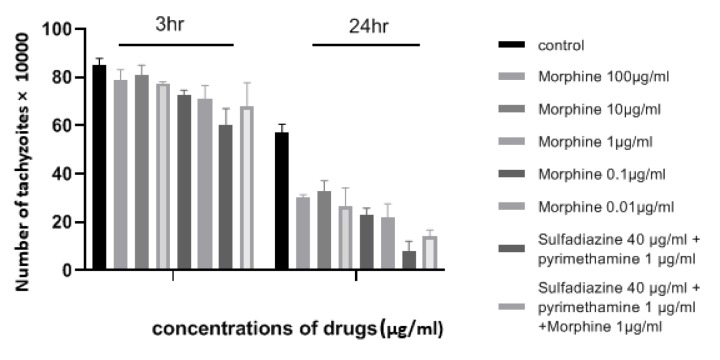
Mean and standard deviation of the number of tachyzoites of *T. gondii* cultured in different concentrations of morphine in 3 h and 24 h compared to control groups (sulfadiazine plus pyrimethamine and untreated). Quantity is ×10^4^.

The number of tachyzoites of *T. gondii* was investigated in the presence or absence of various concentrations of morphine after 3 h and 24 h and a temperature of 24 °C (Supplementary Table 1). The results demonstrated that tachyzoite proliferation decreased significantly in the presence of all concentrations of morphine compared to those exposed to no drugs after 24 h (P < 0.05) (Figure 1(Fig. 1)). Furthermore, it was shown that exposure to different concentrations of morphine for 3 h was not effective and toxic when compared to the negative control. The growth inhibitory effects of the drugs were dose-dependent, such that 0.01 µg/ml and 100 µg/ml concentrations showed the most and the least efficacies, respectively, on inhibiting the proliferation of tachyzoites of *T. gondii*. The greatest differences were related to morphine with concentration of 0.01 μg/ml and sulfadiazine plus pyrimethamine (S+PYR) after 24 h.

### Uninfected macrophage cells viability test

**Figure 2:**
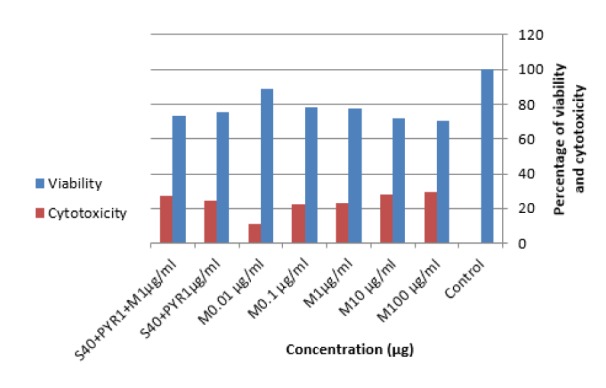
Viability (%) and cytotoxicity of the uninfected RAW macrophages with dissimilar concentrations of drugs and on control group (P < 0.05; ANOVA). M: Morphine, S: Sulfadiazine, PYR: Pyrimethamine

Toxicity effects of morphine on uninfected raw macrophage cells were explored by optical density (OD) following MTT assay to access the best concentration for further experiments. In this regard, the effects of 5 different concentrations of morphine (100, 10, 1, 0.1, and 0.01 μg/ml) were measured after 24 h. According to the data, the percentage viability declined with increases in drug concentrations. On the other hand, the concentration of 100 μg mL^−1^ had the maximum toxicity (Figure 2(Fig. 2)) at concentration of 0.01 μg/mL of morphine; the results were similar to untreated control group. In addition, the viability rate for both sulfadiazine plus pyrimethamine (S+PYR) alone and in combination with morphine (S+PYR+M) were measured as 75.6 % and 73 %, respectively. More details can be found in Supplementary Table 2.

### Flow cytometry analysis

**Figure 3:**
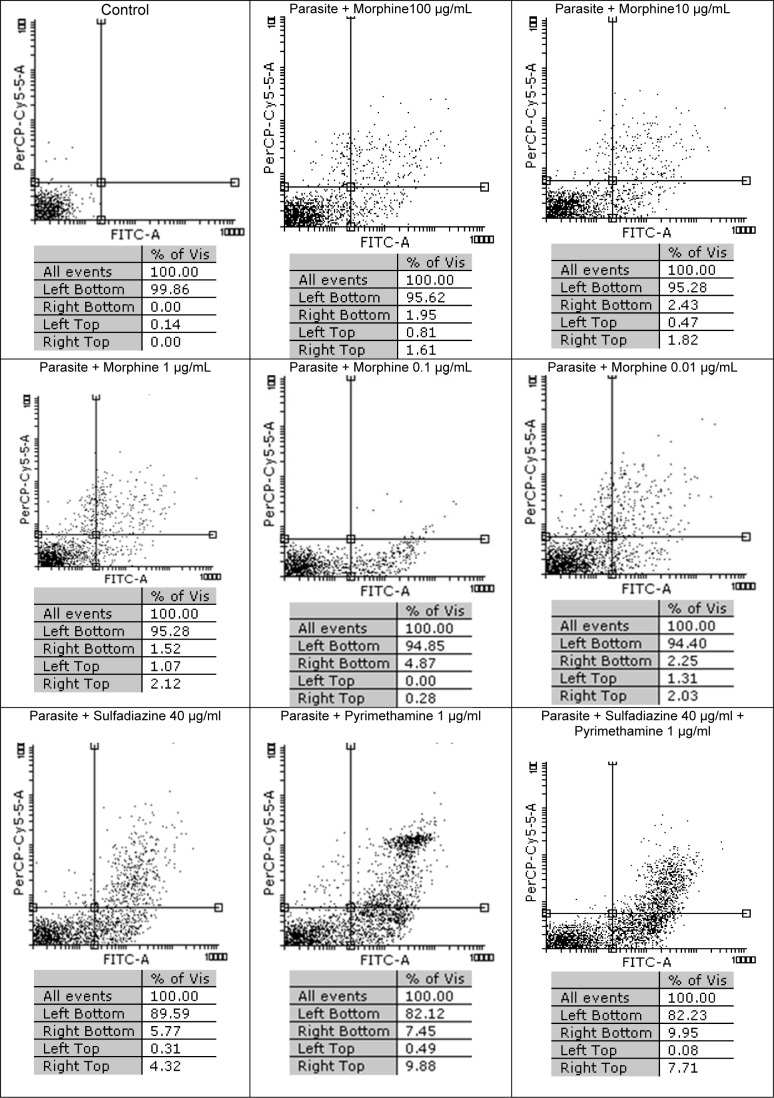
Flow cytometry results of the effect of morphine with dissimilar concentrations on tachyzoites of *T. gondii* viability and comparing them with the control group (untreated) after 3 h. Regions of quadrat show necrosis cells (propidium iodide positive) in left top, late apoptosis in right top, right bottom region belongs to apoptotic cells (annexin positive) and left bottom region belongs to live cells.

**Figure 4:**
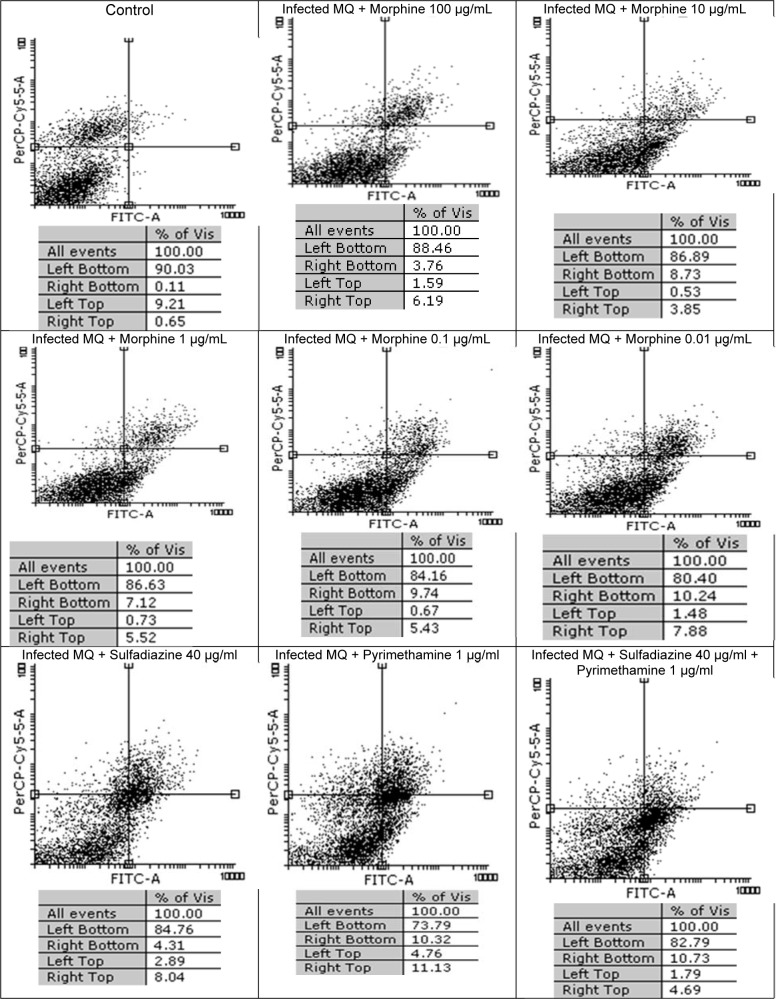
Flow cytometry results of the effect of morphine with 5 different concentrations (100, 10, 1, 0.1 and 0.01 μg/ml) on infected MQs viability and comparing them with the control group (untreated) after 24 h. Regions of quadrat show necrosis cells (propidium iodide positive) in left top, late apoptosis in right top, right bottom region belongs to apoptotic cells (annexin positive) and left bottom region belongs to live cells.

In Figure 3(Fig. 3) the results of flow cytometric analysis for tachyzoites with dissimilar concentrations of morphine and with no drugs are shown. It was observed that the survival rates among five concentrations of morphine (100, 10, 1, 0.1 and 0.01 μg/ml) were 95.62 %, 95.28 %, 95.28 % , 94.85 %, and 94.40 %, respectively, after 3 h of treatment whereas the control group (tachyzoites) without treatment had 99.86 % viable parasites. In groups treated with sulfadiazine (S), pyrimethamine (PYR) and sulfadiazine plus pyrimethamine (S+PYR) demonstrated more lethal effects on tachyzoites of *T. gondii* rate than morphine group. The percentage of apoptosis induced after 24 h in *T. gondii *infected MQs were 5.35 %, 7.85 %, 9.26 %, 10.41 %, and 11.72 %, after being treated with 100, 10, 1, 0.1, and 0.01 µg/ml of morphine, respectively, while in the negative control group (macrophages) was 9.32 % (Figure 4(Fig. 4)). It was found that the maximum toxicity was related to low concentration (0.01 µg/ mL) of morphine. Additionally, the percentage of apoptosis in infected macrophages group treated with sulfadiazine (S), pyrimethamine (PYR) and sulfadiazine plus (S+PYR) pyrimethamine were 7.20 %, 15.08 % and 12.52 %, respectively. This shows that the drugs have no toxic effect on tachyzoites after 3 hours and were not significantly different from control.

### Quantification of parasite load ofmacrophage cells using quantitativereal-time PCR (q-PCR)

The results demonstrate that parasite load was significantly reduced in MQs treated with drugs before infection compared with those in the MQs treated with drugs after infection (p<0.001). However, the parasite load decreased in both groups treated with drugs compared with control group without drugs. Low concentrations (e.g., 0.01 and 0.1 µg/ mL) of morphine were more effective in reducing parasite load than high concentrations (Table 1(Tab. 1)).

### Quantification of parasite load of spleen tissues using quantitative real-time PCR (q-PCR)

The parasite load for all groups was measured by comparison with the standard curve and qPCR analysis. As represented in Table 2(Tab. 2), compared to the untreated group (negative control), a remarkable decrease occurred in the parasite load in the drug groups (P < 0.001). The findings showed that the greatest reduction of parasite load was seen in the group that received morphine before being challenged with parasites.

### Cytokine assay

**Figure 5:**
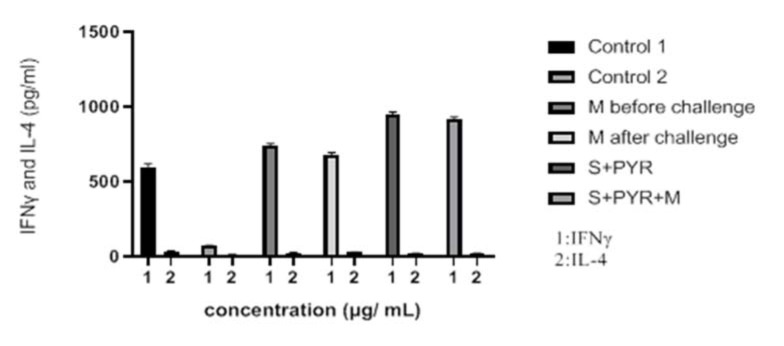
Levels of IFNγ and IL-4 (pg/ml) cytokines secreted from spleen lymphocyte culture in test and control groups after 72 h stimulation with *Toxoplasma* Lyzate Antigen. S: Sulfadiazine (40 mg/kg/day), PYR: Pyrimethamine (1 mg/kg/day), M: Morphine (1 mg/kg, once a week).

IFN-γ and IL-4 cytokine levels after 3 days of exposure in treated and untreated groups were estimated by ELISA. The mean IFN-γ level in the mice that received morphine before challenge was significantly higher than control group (P < 0.05) (Figure 5(Fig. 5)). Considerable differences in IFN-γ cytokine level were observed in the animals that received sulfadiazine plus pyrimethamine (SDZ+PYR) alone and in combination with morphine (M+S+PYR) in comparison with other groups (P < 0.05). It was found that there was no significant difference in IL4 measurements among all groups. On the other hand, the mean IL4 level was similar in the treated and control groups (Table 3(Tab. 3)).

### Survival rate measurement

**Figure 6:**
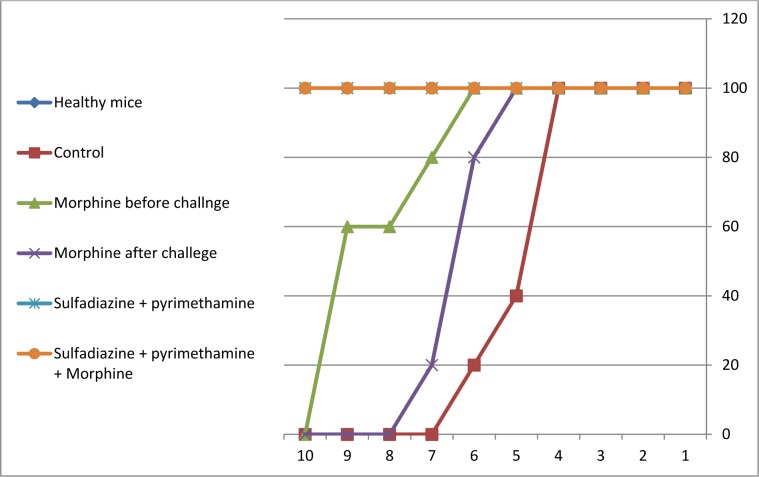
Survival rates of treated and control BALB/c mice during 10 days after and before challenge with 1×10^4^ tachyzoite forms of *T. gondii *RH strain (5 mice per group). One group treated with morphine (1 mg/kg, once a week) for 3 weeks before challenge with 1×10^4^ tachyzoite forms of *T. gondii *RH strain. The rest of the groups after infection treated with morphine alone (1 mg/kg, once a week), sulfadiazine (40 mg/kg/day) pluse pyrimethamine (1 mg/kg/day), and sulfadiazine (40 mg/kg /day) plus pyrimethamine (1 mg/kg /day) in combination with morphine (1 mg/kg, once a week). We used healthy mice as a negative control group, which just received PBS whereas the positive control group was infected with tachyzoites.

For evaluation of survival rate, five mice in each group were treated, then followed for 10 days. Mortality in untreated infected groups started from day 5 after the infection and all mice died by the seventh day of the study. Mortality in the morphine before challenge group started from day 7 after the infection and all mice died by the tenth day of the study. In the morphine after challenge group, the mortality of mice began from day 6 and all mice died by the eighth day. In addition, there were no deaths in animals exposed to the sulfadiazine plus pyrimethamine (S+PYR) alone and in combination with morphine (M+S+PYR). The survival rates of the mice in the treatment and control groups are illustrated in Figure 6(Fig. 6).

## Discussion

Despite that many effective prevention and treatment strategies exist, toxoplasmosis remains an enormous threat that unfortunately lacks a global solution. Lack of sufficient budget, unacceptable toxicity, and several side effects of medicines have been identified as major obstacles in achieving therapeutic goals (Maubon et al., 2010[17]; Pinzan et al., 2015[21]). Potent new drugs are therefore required to improve early treatment against toxoplasmosis.

In this study, we evaluated the preventive and therapeutic effects of morphine on the tachyzoites of *Toxoplasma gondii* *in vitro *and* in vivo*. It is known that the use of opiates such as morphine, apart from being analgesics, can have effects on both innate and cell-mediated immunity and stimulate the immune responses on different parasitic infections (Liang et al., 2016[16]; Sharp, 2004[24]). Consequently, the use of morphine, because of its therapeutic implications, has become popular in recent years. Opiates have been shown to modify immune responses directly with the help of opioid receptors on immune cells, while they can act indirectly through opioid receptors in the central nervous system (CNS) (Salimi et al., 2013[23]; Singal and Singh, 2005[27]). It should be noted, following morphine binding to opioid receptors, with nitric oxide released and vasodilation promoted reduce the healing process in cutaneous leishmaniasis (Alavi-Naini, 2008[1]).

Nevertheless, several publications have confirmed that prolonged use of morphine attenuates the general functions of the immune cells including macrophages and polymorphonuclear leukocytes resulting in suppression of the immune system and increased susceptibility to a variety of infectious diseases (Friedman et al., 2006[11]; Risdahl et al., 1993[22]; Wang et al., 2005[29]). 

The results of the current study revealed that morphine inhibited the proliferation of tachyzoites of *T. gondii* in a concentration- and time-dependent manner and provided the best result after 24 hours for all concentrations. Notably, a morphine concentration of 0.01 µg/mL was most effective in inhibiting tachyzoites growth after 24 h incubation. In a previous study of promastigotes of *Leishmania major*, the results were similar (Jabari et al., 2019[14]).

Sulfadiazine plus pyrimethamine in combination with morphine was also effective in reducing tachyzoites numbers like the reference drug (S+PYR). It was found that the viability of macrophage cells exposed to morphine was also to be based on a dose-dependent response. The results of MTT showed that as concentration of morphine was increased, uninfected macrophage cell viability was reduced. The demonstration of apoptosis and necrosis by flow cytometry has been previously verified in uninfected MQs with different morphine concentrations and the results showed low apoptosis and necrosis (Ebrahimisadr et al., 2018[6]). Also, morphine was able to induce little apoptosis in parasite-infected MQs. Compared to the control group (MQs without treatment) induction of necrosis was reduced in infected MQs during treatment with morphine. Moreover, apoptosis occurred in tachyzoites of *T. gondii* after exposure with different morphine concentrations, although these apoptotic effects were not significant. Morphine showed lower toxicity upon infected MQs and parasite compared with sulfadiazine (S), pyrimethamine (PYR) and sulfadiazine plus pyrimethamine (S+PYR) groups. Hence, it can be assumed that morphine may act as an appropriate anti-*Toxoplasma *agent through amplification of the immune response and expression of cell level receptors. With regard to the preventive and/or adjuvant role of morphine, when the MQs were treated before tachyzoite infection *in vitro*, a significant reduction was observed in parasite burden. It was also shown that the antiparasite behavior of morphine was dose-dependent. Interestingly, the lowest parasite burden was at a concentration of 0.01 μg/mL morphine. MQs are essential for generation of numerous immune responses by enhanced phagocytosis and secrete particular cytokines in combatting toxoplasmosis. Also, MQs are a significant site of action for opioid drugs (Cabral et al., 2018[3]; Ebrahimisadr et al., 2018[6]; Pacifici et al., 1994[20]). Therefore, it can be concluded that morphine can induce a strong immune response with enhancing defensive function of the MQs as well as inflammatory cytokines to confine this parasitic disease. The results obtained from the murine model demonstrated that the parasitic burden was decreased significantly in mice treated with morphine but this reduction in those received morphine before challenge was more specific. Also, a considerable reduction in the parasitic load was observed in groups treated with sulfadiazine plus pyrimethamine alone or in combination with morphine. There was significant relationship between survival rate and parasitic burden. The mortality rate in groups that received morphine before parasitic challenge was lower than those received morphine after parasitic challenge. In the context of the toxoplasmosis in mouse model, production of IFN-γ from immune system cells as the adaptive cellular immune response has a substantial role in the controlling and restricting growth of the parasite and also induce a strong Th1 type immune response with the help of MQs (Foroutan et al., 2019[10]; Krishnamurthy et al., 2017[15]). Levels of interferon-gamma (IFNγ) production were high in mice treated with morphine before infection (just like the sulfadiazine plus pyrimethamine) compared with those in the no drug group, whereas levels of interleukin 4 (IL-4) productions were low across all treatment groups. Hence, it can be inferred that the morphine through boosting the immune system reduces disease due to *T. gondii *in BALB/c mice.

However, the presented results demonstrate that morphine has an acceptable ability to treat infection due to *Toxoplasma gondii* under both *in vitro* and *in vivo* conditions. 

## Conclusion

Based on the results of *in vitro* and *in vivo* conditions, antiparasitic activity of morphine on tachyzoites of *Toxoplasma gondii* and infected macrophages was promising. Also, morphine in pretreated mice showed higher anti-Toxoplasma behavior than morphine in posttreated mice. On the other hand, the preventive effects of morphine were stronger than the therapeutic effects and therefore, morphine can be used as an adjunctive treatment at low doses for the treatment of toxoplasmosis. Furthermore, the efficiency of morphine is increased when it is used in combination therapy with sulfadiazine plus pyrimethamine (S+PYR). However, the results of the study suggest that morphine can be considered as an additional drug either alone or along with other anti-Toxoplasma products for treatment in future studies.

## Acknowledgement

The authors would like to thank all staff of Department of Parasitology of Tarbiat Modares University, Iran. This paper is issued from thesis of Leila Zaki, Ph.D student of Medical Parasitology.

## Ethical statement

This experiment was approved by the Research Department of Medical Sciences, Tarbiat Modares University, with issue number 52D/1883 and approved by the Ethical Committee with issue number 52D/1239.

## Conflict of interest

The authors declare that they have no conflict of interest.

## Supplementary Material

Supplementary information

## Figures and Tables

**Table 1:**
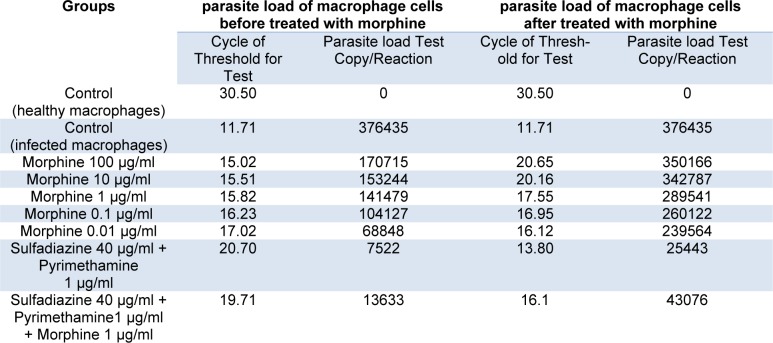
Cycle of Threshold (CT) and parasite load test copy/reaction according to Real Time PCR method for macrophage cells before and after challenge with 1 × 10^4^ tachyzoite forms of *T. gondii *RH strain and control groups.

**Table 2:**
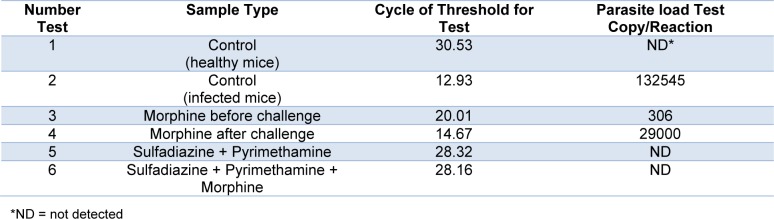
Cycle of Threshold (CT) and parasite load test copy/reaction according to Real Time PCR method for all treated and control groups

**Table 3:**
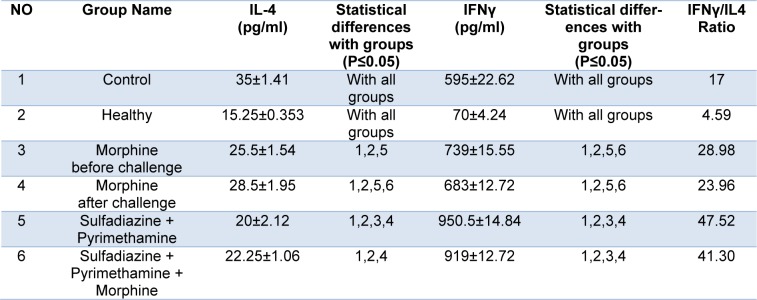
Mean and standard deviation of interleukin 4 (IL-4) and interferon gamma (IFNγ) concentrations (pg/ml) and IFNγ/IL4 ratio in treated and control groups
