# Supplementary material for: Toxoplasma gondii: Preventive and therapeutic effects of morphine and evaluation of treatment parameters of tachyzoites and infected macrophages in vitro and in a murine model
Source: EXCLI J. 2020 Apr 20;19:514–27. doi: 10.17179/excli2019-1961 (PMC7214776; doi:10.17179/excli2019-1961)
Supplement: Supplementary information [file EXCLI-19-514-s-001.pdf]

EXCLI Journal 2020;19:514-527 – ISSN 1611-2156
Received: November 06, 2019, accepted: April 16, 2020, published: April 20, 2020

Supplementary information to:

TOXOPLASMA GONDII: PREVENTIVE AND THERAPEUTIC EFFECTS OF MORPHINE AND EVALUATION OF TREATMENT PARAMETERS OF TACHYZOITES AND INFECTED MACROPHAGES *IN VITRO* AND IN A MURINE MODEL

Leila Zaki[1], Fatemeh Ghaffarifar[1*], Zohreh Sharifi[2], John Horton[3], Javid Sadraei[1]

1 Department of Parasitology, Faculty of Medical Sciences, Tarbiat Modares University, Tehran, Iran
2 Blood Transfusion Research Center, High Institute for Research and Education in Transfusion Medicine, Tehran, Iran
3 Tropical Projects, Hitchin, United Kingdom

* **Corresponding author:** Fatemeh Ghaffarifar, Ph.D., Department of Parasitology, Faculty of Medical Sciences, Tarbiat Modares University, Tehran, P.O. Box 14115-111, Iran, Tel: +98-21-82884553, Fax: +98-21-82884555, E-mail: ghafarif@modares.ac.ir

http://dx.doi.org/10.17179/excli2019-1961

This is an Open Access article distributed under the terms of the Creative Commons Attribution License (http://creativecommons.org/licenses/by/4.0/).

EXCLI Journal 2020;19:514-527 – ISSN 1611-2156
Received: November 06, 2019, accepted: April 16, 2020, published: April 20, 2020

Supplementary Table 1: The Mean and SD of the tachyzoites number influenced by various concentrations of morphine and control group after 3 and 24 hours

Time / Groups	3 h (×10^4)	24 h (×10^4)
	Mean ± SD	Mean ± SD
Control	85±2.8	57±3.5
Morphine 100 µg/ml	79±4.2	30±1.4*
Morphine 10 µg/ml	81±4	33±4.2*
Morphine 1 µg/ml	77.5±0.7	26.5±7.7*
Morphine 0.1 µg/ml	72.5±2.1	23±2.8*
Morphine 0.01 µg/ml	71±5.6	22±5.6*
Sulfadiazine 40 µg/ml + pyrimethamine 1 µg/ml	60±7*	8±1.4*
Sulfadiazine 40 µg/ml + pyrimethamine 1 µg/ml + Morphine 1 µg/ml	68±9.8	14±2.8*

Supplementary Table 2: Viability of drug-treated macrophages in comparison with the control group using MTT

Name of drugs / (µg/ml)	Viability (%)	Cytotoxicity
Control	100	0
Morphine 100 µg/ml	70.3	29.7
Morphine 10 µg/ml	72.1	27.9
Morphine 1 µg/ml	77.2	22.8
Morphine 0.1 µg/ml	77.9	22.1
Morphine 0.01 µg/ml	88.8	11.2
Sulfadiazine 40 µg/ml + Pyrimethamine 1 µg/ml	75.6	24.4
Sulfadiazine 40 µg/ml + Pyrimethamine 1 µg/ml + Morphine 1 µg/ml	73	27
